# Differential acute impact of therapeutically effective and overdose concentrations of lithium on human neuronal single cell and network function

**DOI:** 10.1038/s41398-021-01399-3

**Published:** 2021-05-12

**Authors:** Julia Izsak, Henrik Seth, Margarita Iljin, Stephan Theiss, Hans Ågren, Keiko Funa, Ludwig Aigner, Eric Hanse, Sebastian Illes

**Affiliations:** 1 Institute of Neuroscience and Physiology, Sahlgrenska Academy at University of Gothenburg, Gothenburg, Sweden; 2 Institute of Clinical Neuroscience and Medical Psychology, Medical Faculty, Heinrich Heine University, Düsseldorf, Germany; 3 Result Medical GmbH, Düsseldorf, Germany; 4 Institute of Neuroscience and Physiology, Section of Psychiatry and Neurochemistry, Sahlgrenska Academy at University of Gothenburg, Gothenburg, Sweden; 5 Sahlgrenska Cancer Center, Institute of Biomedicine, Sahlgrenska Academy at University of Gothenburg, Gothenburg, Sweden; 6 Oncology Laboratory, Department of Pathology, Sahlgrenska University Hospital, Gothenburg, Sweden; 7 Institute of Molecular Regenerative Medicine, Spinal Cord Injury and Tissue Regeneration Center Salzburg, Paracelsus Medical University, Salzburg, Austria

**Keywords:** Stem cells, Molecular neuroscience

## Abstract

Lithium salts are used as mood-balancing medication prescribed to patients suffering from neuropsychiatric disorders, such as bipolar disorder and major depressive disorder. Lithium salts cross the blood-brain barrier and reach the brain parenchyma within few hours after oral application, however, how lithium influences directly human neuronal function is unknown. We applied patch–clamp and microelectrode array technology on human induced pluripotent stem cell (iPSC)-derived cortical neurons acutely exposed to therapeutic (<1 mM) and overdose concentrations (>1 mM) of lithium chloride (LiCl) to assess how therapeutically effective and overdose concentrations of LiCl directly influence human neuronal electrophysiological function at the synapse, single-cell, and neuronal network level. We describe that human iPSC-cortical neurons exposed to lithium showed an increased neuronal activity under all tested concentrations. Furthermore, we reveal a lithium-induced, concentration-dependent, transition of regular synchronous neuronal network activity using therapeutically effective concentration (<1 mM LiCl) to epileptiform-like neuronal discharges using overdose concentration (>1 mM LiCl). The overdose concentration lithium-induced epileptiform-like activity was similar to the epileptiform-like activity caused by the GABA_A_-receptor antagonist. Patch–clamp recordings reveal that lithium reduces action potential threshold at all concentrations, however, only overdose concentration causes increased frequency of spontaneous AMPA-receptor mediated transmission. By applying the AMPA-receptor antagonist and anti-epileptic drug Perampanel, we demonstrate that Perampanel suppresses lithium-induced epileptiform-like activity in human cortical neurons. We provide insights in how therapeutically effective and overdose concentration of lithium directly influences human neuronal function at synapse, a single neuron, and neuronal network levels. Furthermore, we provide evidence that Perampanel suppresses pathological neuronal discharges caused by overdose concentrations of lithium in human neurons.

## Introduction

Lithium salts are used as mood-balancing medication prescribed to patients suffering from neuropsychiatric disorders, such as bipolar disorder and major depressive disorder^1^. After oral application of lithium, lithium is taken up by the gut system, becomes distributed within the blood system, crosses the blood-brain barrier, thereby, lithium is present within the brain parenchyma^2^, and most likely influences human neuronal function within less than a few hours after oral application. Thus, the functional alterations related to acute exposure to lithium at individual human neuron as well as neuronal circuit level represents an interesting but unaddressed question.

In addition to the beneficial mood-balancing effects, lithium medication may also cause overdose symptoms, e.g., seizures (e.g., ref. ^3^, in lithium-treated patients). Since the risk of lithium-induced seizures, among other severe symptoms, is substantial in patients treated with a high concentration of lithium (>1.2 mM), co-medication of antiepileptic drugs might reduce the risk of lithium-induced seizures. Testing co-medication approaches of increased concentrations of lithium with antiepileptic drugs in humans is not feasible. Therefore, a preclinical human model system with lithium-induced abnormal neuronal activity, that is allowing the evaluation of the efficacy of drugs in counteracting pathophysiological neuronal discharges is an unmet medical research need.

The introduction of induced pluripotent stem cell (iPSC) technology^4^ made it possible to generate human neurons and astrocytes from healthy and morbid human individuals^5–7^. Human iPSC-neuronal models have been used to describe the long-term effects of lithium on the function of human iPSC neurons^8,9^. However, if and how therapeutically effective (<1.2 mM) and overdose concentrations (>1.2 mM) of lithium rapidly (within minutes) influence human synaptic, single neuron, and neuronal network function, as occur shortly after oral application of lithium in patients, has not been described.

As described in a previous study of ours^10^, we are combining microelectrode array (MEA) technology with human iPSC-cortical neurons to assess human neuronal network function. Here, human iPSC-cortical neurons and astrocytes are cultured on an array of electrodes embedded in a glass surface. MEA recordings are non-invasive and allow the measurements of action potentials (APs) (also referred to as spikes) generated by neurons in close vicinity to the extracellular recording electrode. As described previously, we have developed a robust procedure where human iPSC-cortical neurons mature and generate population-wide bursting, i.e., the synchronous activity of neurons, within 3 weeks in vitro. Thus, such human neuronal sensor chips provide valuable information about physiological and pathophysiological neuronal network function. In this study, we determine that there are differences in neuronal network activity pattern of human iPSC-cortical neurons exposed to therapeutically effective (<1 mM) and overdose concentrations (>1 mM) of lithium chloride. In addition, patch–clamp recordings of human neurons exposed to lithium provide mechanistic insights on how lithium directly influences the synaptic function, passive membrane properties and excitability of human cortical neurons. The combined MEA and patch–clamp data sets provide the rationale to select and evaluate Perampanel—the only clinically used AMPA-receptor antagonist—as a prospective drug to suppress lithium-overdose mediated epileptiform-like activity in human iPSC-cortical neurons and neuronal networks.

## Materials and methods

### Ethical statement

We confirm that all methods were carried out in accordance with relevant guidelines and regulations. We confirm that all experimental protocols were approved by the named institutions. Informed consent was obtained from all subjects. Work with human iPSC lines was approved by a local ethics committee (Regionala etikprövningsnämnden i Göteborg, with the ethical approval number: DNR 172-08).

### Cultivation, neural differentiation of iPSC lines, and 3D neural aggregate formation

All iPSC lines were cultured under feeder-free conditions in Cellartis DEF-CS^™^ (Takara Bio Europe AB) or mTESR1 (StemCellTechnologies) at 37 °C in a humidified atmosphere of 5% CO_2_ in the air. Three in-lab produced human iPSC lines^11^ were used for neural induction by applying the DUAL-SMAD inhibition protocol^12^ and frozen stocks of human iPSC-neural stem cells (hiPSC-NSC) were obtained according to our previously published procedure^10^. Frozen stocks of hiPSC-NSC were thawed and cultured in neural culture media on Poly-l-Ornithine (PLO)/Laminin-coated 3.5 cm culture plates. Neural culture media consisted of DMEM/F12 GlutaMAX, Neurobasal, N2 supplement, B27 supplement, 5 µg/ml insulin, 1 mM Ultra glutamine, 100 µM nonessential amino acids, 100 µM 2-mercaptoethanol, 50 U/ml penicillin, and streptomycin. After 7–10 days, 3D-neural aggregates with diameters ≤ 150 µm were manually transferred on PLO/laminin-coated coverslips or MEAs. For neuronal differentiation, BrainPhys-media supplemented with N2 supplement, B27 with vitamin A, 2 mM Ultra glutamine, 50 U/ml Pen/Strep, and 200 µM ascorbic acid were used. The media was further supplemented with neurotrophic factors: brain-derived neurotrophic factor, glial-derived neurotrophic factor, transforming growth factor-β (TGF-β) [20 ng/ml each], and optional DAPT [10 µM]. Half media exchanges were performed twice a week. For further information about used culture media and procedures see elsewhere^10^.

### Immunocytochemistry and confocal imaging

For the immunocytochemical characterization of the model system, hiPSC-3D neural aggregates were seeded and cultured on PLO/laminin-coated glass coverslips or 96-well plates (Greiner) for 14–21 days in BrainPhys-media with supplements (described above). The cultures were then washed in phosphate-buffered saline (PBS), pH 7.2, and fixed for 20 min in 4% paraformaldehyde at room temperature. After fixation, the cells were incubated with 1% bovine serum albumin for 30 min. Primary antibodies binding to neuronal (bIIITub, MAP2ab, TBR1, CTIP2, SATB2, and Parvalbumin-PV), astrocytic (S100 β), and synaptic (PSD95, VGlut1) structures were diluted in blocking solution with 0.025% Triton-X and were applied at 4 °C overnight. After washing in PBS, appropriate secondary antibodies together with DAPI nuclear counterstaining were applied for 2 h at room temperature. Images were collected with a confocal-laser scanning microscope (LSM 700 META Zeiss). The used primary and secondary antibodies are summarized in Supplementary Table 1.

### Quantification of cortical sublayer-specific neurons

Human iPSC-derived 3D neural aggregate (3DNA) cultures were generated from two individual human iPSC lines as described before (C2, C3; for the origin of human iPSC lines see elsewhere^11^) and were cultured on PDL/Laminin-coated 96-well plates. After 20–21 days in vitro, cultures were PFA-fixated and stained with MAP2ab combined either with TBR1, CTIP2, or SATB2 primary antibodies and corresponding secondary fluorescent antibodies, as described before. By using a 40×-water immersion objective, 5 µm optical slices were acquired with a confocal laser scanning microscope (LSM 700 META Zeiss). Images were taken from three individual cultures where the top area of 20 3DNAs and 20 areas outside of 3 DNAs per cell line were acquired. Quantification was conducted by high content analysis workflow using Cell Profiler 4.07 and Cell Profiler analyst 2.2.1. Quantification masks were generated to detect and count the number of MAP2ab-positive neurons that have a nuclear TBR1, CTIP2, or SATB2 signal.

### Multi-electrode array recordings and pharmacological experiments

Four to eight hiPSC-3D-neural aggregates were seeded as a 5 µl drop directly on PLO/biolaminin 521 coated electrode arrays of 6-well MEAs. After 1 h, BrainPhys^TM^ media with supplements were added. Half media exchanges were performed twice a week. MEAs had Ti/TiAu electrodes with PEDOT-CNT (carbon nanotube poly-3,4-ethylene-dioxythiophene) of 30 µm diameter and 200 µm spacing. Electrode configuration was nine recording electrodes per well in the six-well MEAs. MEA-electrodes had an input impedance of 30–50 kΩ according to the specifications of the manufacturer (Multi Channel Systems). The recording sampling rate was 25 kHz on all MEA electrodes. MC_Rack (Multi Channel Systems) was used to visualize and store MEA data. Offline-spike detection was performed by the SPANNER software suite (RESULT Medical; see also ref. ^13^). Synchronous network activity was analyzed by population burst detection using custom-built Matlab software (see ref. ^14^). For pharmacological experiments, cultivation media was removed and fresh BrainPhys media without any supplements was added (300 µl per well of 6-well MEA). BrainPhys media was continuously gassed with a gas containing 95% O_2_ and 5% CO_2_. Lithium chloride, sodium chloride, picrotoxin, gabazine or dimethyl sulfoxide (DMSO), respectively, were solved in BrainPhys media before use. Perampanel powder provided by EISAI was solved in DMSO with a stock concentration of 10 mM. Pre-diluted aliquots of 1 mM and 100 µM Perampanel were prepared in BrainPhys media. After the application of drugs, at least 16 min of consecutive 2 min recordings were performed, where only the last 10 min were used for data analysis. To assess the effective concentration of a GABA_A_-receptor antagonist, a pilot experiment (*n* = 3) was conducted, which revealed that concentrations >10 µM of PTX induce a clear increase in the number of population bursts (Supplementary Fig. 10).

### Patch–clamp recordings

Five to ten hiPSC 3D-neural aggregates were seeded on PLO/biolaminin 521 coated coverslips and cultured with BrainPhys media with supplements. Half media exchanges were performed twice a week. After 20–40 days in culture, patch–clamp recordings were performed. The coverslips were mounted under a differential interference microscope (Nikon E600FN) together with a CCD camera (Sony XC-73CE) to visually identify the cells and to visualize the recording electrode connected to the neuron via a borosilicate glass micropipette (resistance 3–6 MΩ). Cells were perfused (2–3 ml/min) with artificial CSF (ASCF) containing: 1 mM NaH_2_PO_4_, 123 mM NaCl, 26 mM NaHCO_3_, 3 mM KCl, 1 mM MgCl_2_, 2 mM CaCl_2_, and 10 mM d-glucose. The micropipette was filled with an intracellular solution containing; 127 mM K-gluconate, 8 mM KCl, 10 mM HEPES, 15 mM phosphocreatine, 4 mM Mg-ATP, 0.3 mM Na-GTP (pH ~7.3 and osmolality 280–300 mOsm). Patch–clamp recordings were performed on cells at the edge of 3D-neural aggregates visually identified using infrared differential interference contrast video microscopy. The data were collected with a sampling frequency of 10 kHz and filtered at 3 kHz by an EPC-9 amplifier (HEKA Elektronik, D-67466 Lambrecht/Pfalz, Germany). After opening, the cell was allowed to rest for 5 min before recordings started. Series resistance was monitored using a 20 ms 10 mV hyperpolarizing pulse. The series resistance was not allowed to exceed 20 MΩ in whole-cell recordings, or to change more than 20% during an experiment, otherwise, the experiment was discarded. Whole-cell recordings were carried out at 32 °C.

We first recorded the firing response, at baseline conditions, to step-wise current injections (300 ms) in whole-cell current–clamp and spontaneous synaptic activity (i.e., excitatory postsynaptic currents (EPSCs) and inhibitory postsynaptic currents (IPSCs)) in whole-cell voltage–clamp. We then repeated this protocol in an ACSF containing first 1 mM LiCl then 5 mM LiCl. To control for the effects of time on the recordings we repeated the protocol once again after wash-out of LiCl for 10 min.

For spontaneous synaptic activity cells were clamped at −70 mV for recordings of the α-amino-3-hydroxy-5-methyl-4-Isoxazolepropionic acid receptor (AMPAR)-mediated EPSCs and at 0 mV for recordings of the γ-aminobutyric acid receptor (GABAR)-mediated IPSCs.

Spontaneous synaptic activity, i.e., frequency and amplitude, was analyzed in Minianalysis 6.0.3 (Synaptosoft, Fort Lee, NJ, USA). Input resistance was calculated using Ohm’s law (*U* = *R* × *I*) after injecting a 50 pA hyperpolarizing current in the current–clamp. To calculate the AP threshold, we constructed phase-plane plots by plotting the first derivative (i.e., the rate of change) of the membrane potential during the first AP, at current injection, against the membrane potential (Fig. 3C). These plots visualize some aspects of the AP very clearly (i.e., threshold de- and repolarization as well as amplitude). The threshold was then estimated at a derivative (d*V*/d*t*) of 10 mV/ms. Calculations and data analysis were performed in custom-made IGOR Pro 8 (WaveMetrics, Lake Oswego, OR, USA) software.

### Statistical analysis

For statistical analysis, GraphPad Prism (version 7) was used. One-way ANOVA with Dunnett’s correction (baseline compared to the indicated group), One-way ANOVA with Tukey’s correction (comparison between the indicated groups) or two-tailed, unpaired/paired *t* test was used to calculate the shown *p* values. For the visualization of dose–response curves and determination of IC_50_, nonlinear regression analysis was performed. The presented data show mean value ± standard deviation (SD) or mean value ± standard error of the mean, *n* refers to the number of individual cultures and *N* refers to the number of individual experiments.

## Results

### Therapeutic and overdose lithium concentrations cause different neuronal network activity patterns

We applied the commonly used dual-SMAD inhibition protocol^12^ to neural differentiate human iPSCs into telencephalic neural stem cells, which gave rise to hiPSC-derived 3D neural aggregates (3DNA) (Supplementary Fig. 1A–C, for detailed characterization see ref. ^10^). Human 3 DNAs comprised astrocytes and synaptically interconnected neurons that had PSD-95^+^-/vGlut1^+^-synapses (Supplementary Fig. 1C), and formed a functional neuronal network that either showed partial synchronous or synchronous neuronal activity within 2–3 weeks in vitro (Supplementary Fig. 1D). For the discrimination of partial synchronous and synchronous networks a cut-off value of 20% of spikes organized in population bursts (PB) and a population firing rate of 100 spikes per second was used. Partial synchronous neuronal network activity was characterized by less than 20% of spikes organized in PB and these PB had a population firing rate lower than 100 spikes per second (Supplementary Fig. 1D, i). Synchronous neuronal network activity was characterized by more than 20% of spikes that were organized in PB and these PB had a population firing rate higher than 100 spikes per second (Supplementary Fig. 1D, ii). Further immunocytochemical characterization using neuronal marker MAP2ab combined either with cortical layer-specific markers TBR1, CTIP2, SATB2, or inhibitory neuronal marker Parvalbumin (PV) showed that human iPSC-derived 3DNA cultures consist of cortical layer-specific excitatory neurons, predominantly, and few PV^+^ interneurons (Supplementary Fig. 2).

**Fig. 1:**
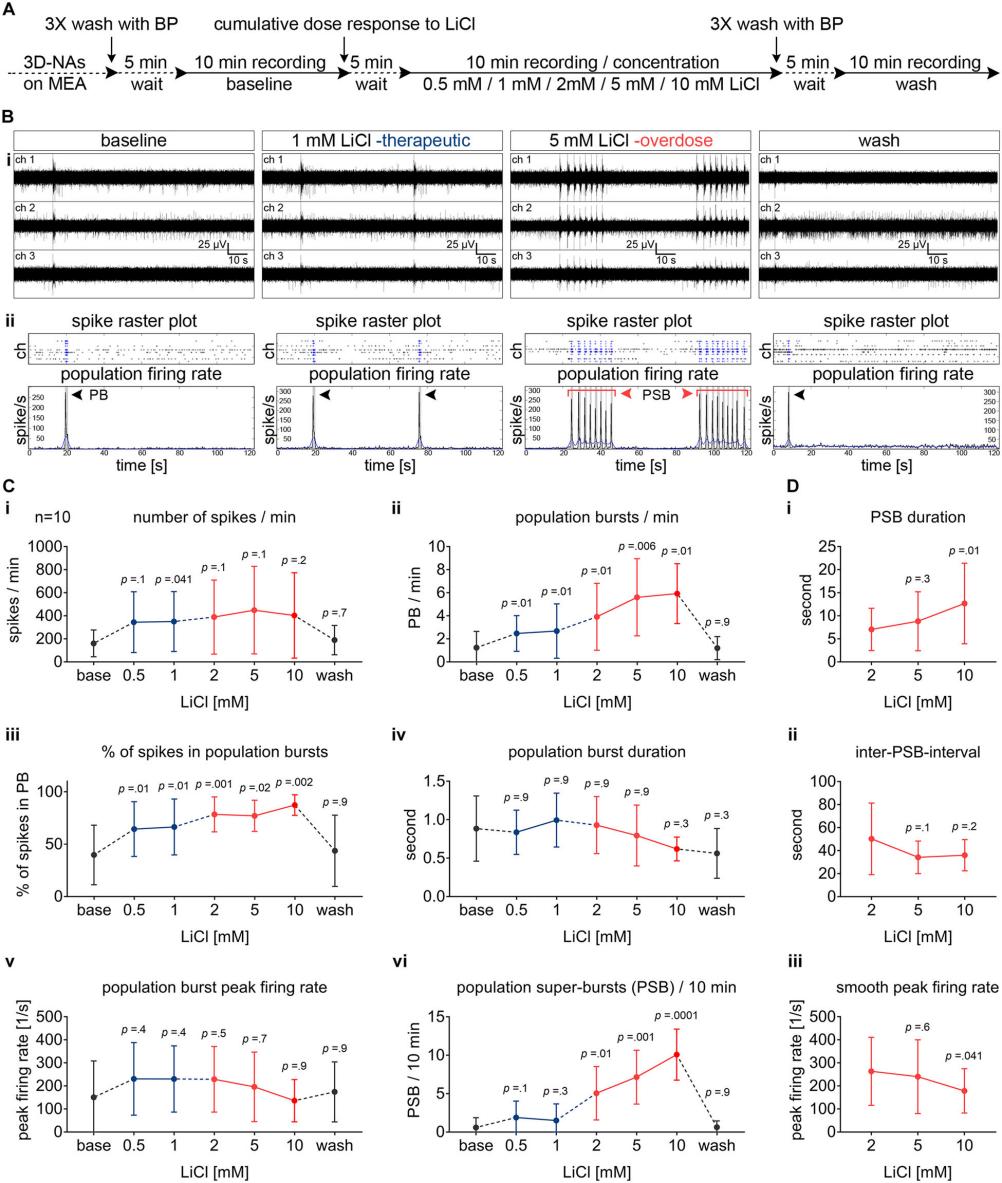
Concentration-dependent bi-modulatory impact of lithium chloride (LiCl) on human cortical networks recorded by MEAs. **A** Schematic representation of the applied protocol to evaluate the cumulative dose–response to LiCl in the hiPSC-derived 3D-neural aggregate cultures with synchronous network activity (day 20–50 after seeding). **B**, **i** Representative examples of MEA-recordings (three individual channels are shown) with their corresponding **B**, **ii** Spike raster plot and population firing rate diagrams showing the properties of network activity in baseline and in response to therapeutic and overdose concentrations of LiCl. The black arrows are marking the population bursts (PB) and the red arrows with the red line mark the population super-bursts (PSB). **C** Diagrams illustrating the change of neuronal network parameters in response to increasing concentrations of LiCl in synchronously active human neuronal networks. **D** The properties of PSB activity in overdose concentrations of LiCl. The data are presented as mean ± standard deviation, *n* = 10, *N* = 3. One-way ANOVA with Dunnett’s correction (baseline compared to the indicated group) was applied to calculate the shown *p* values.

Since the therapeutically effective concentration of lithium lies between 0.5 and 1.2 mM^1,3,15^, we exposed human neurons cultured on MEAs to different therapeutic (≤1 mM, LiCl_thera_) and overdose (>1 mM, LiCl_overd._) concentrations of lithium. MEA recordings were used to describe the acute impact of the different lithium concentrations on human neuronal function at the network level. Baseline activity was recorded in BrainPhys^™^ media, of hiPSC-derived cortical cultures obtained from three different donor-derived iPSC lines. After baseline activity was measured, we consecutively applied increasing concentrations of LiCl followed by three wash-out steps to evaluate if the induced LiCl impact was reversible (Fig. 1A, B).

We observed that acute exposure of human neurons to therapeutically effective concentrations of LiCl_thera_ (up to 1 mM) increased the number of PB (Fig. 1C, ii) and percentages of spikes organized in PB (Fig. 1C, iii), while the number of recorded spikes showed either a nonsignificant or only a minor increase (Fig. 1C, i). Parameters describing the individual PB, such as peak firing rate (Fig. 1C, v) and PB duration (Fig. 1C, iv), did not change when switching from baseline toward therapeutic concentration of LiCl. Thus, parameters describing neuronal network properties rather than global neuronal population activity are suitable to reveal how therapeutically effective concentrations of lithium change the neuronal network pattern of human neurons.

Exposing human neurons to overdose concentrations of LiCl (>1 mM) dramatically changed the network firing behavior in human neuronal networks (Fig. 1).

In synchronously active neuronal networks, we observed that individual single PB events transformed into sequences of several consecutive PB that persisted for several seconds followed by a longer period of neuronal inactivity (Fig. 1B). We termed these long-lasting synchronous events LiCl_overd._-induced population super bursting (PSB). Interestingly, human neuronal networks exposed to overdose concentrations of LiCl showed PSB, more often than what was detected in LiCl untreated and LiCl_thera_-treated human neuronal networks (Fig. 1C, vi). With increasing concentrations of LiCl, the number (Fig. 1C, vi) and duration of PSB (Fig. 1D, i) increased, while the inter-population super bursting interval (Fig. 1D, ii) and smooth peak firing rate (Fig. 1D, iii) decreased. Even though not significantly, we observed that individual PB recorded under overdose concentrations of LiCl showed a LiCl_overd._-dose-dependent reduction of PB duration (Fig. 1C, iv), and peak firing rate (Fig. 1C, v). By assessing neuronal network responses of human neuronal networks exposed to individual LiCl_thera_- and LiCl_overd_-concentrations, we confirmed that the above described LiCl-induced alteration of human neuronal network activity is specific for each applied LiCl concentration and is reversible after washout (Supplementary Figs. 3 and 4). In general, we did not observe a further significant enhancement of population super bursting or changes in properties of population super bursting between population super bursting induced by 5 or 10 mM LiCl.

Similar to synchronously active human neuronal networks, overdose concentration of LiCl altered the neuronal network activity pattern rather than the global neuronal population activity in partial synchronous networks. Partially synchronously active human neuronal networks transformed into highly synchronous neuronal networks (Supplementary Fig. 5A) characterized by concentration-dependent (2, 5, 10 mM) increase in number of PB, percentage of spikes organized in PB, and decrease of the inter-PB interval (Supplementary Fig. 5B).

To summarize, acute application of overdose concentration of Lithium causes abnormal neuronal network activity patterns in human neuronal circuits, which are not present after application of therapeutically effective concentrations of Lithium.

### Lithium overdose-concentration causes epileptiform-like activity in human cortical neurons

**Fig. 2:**
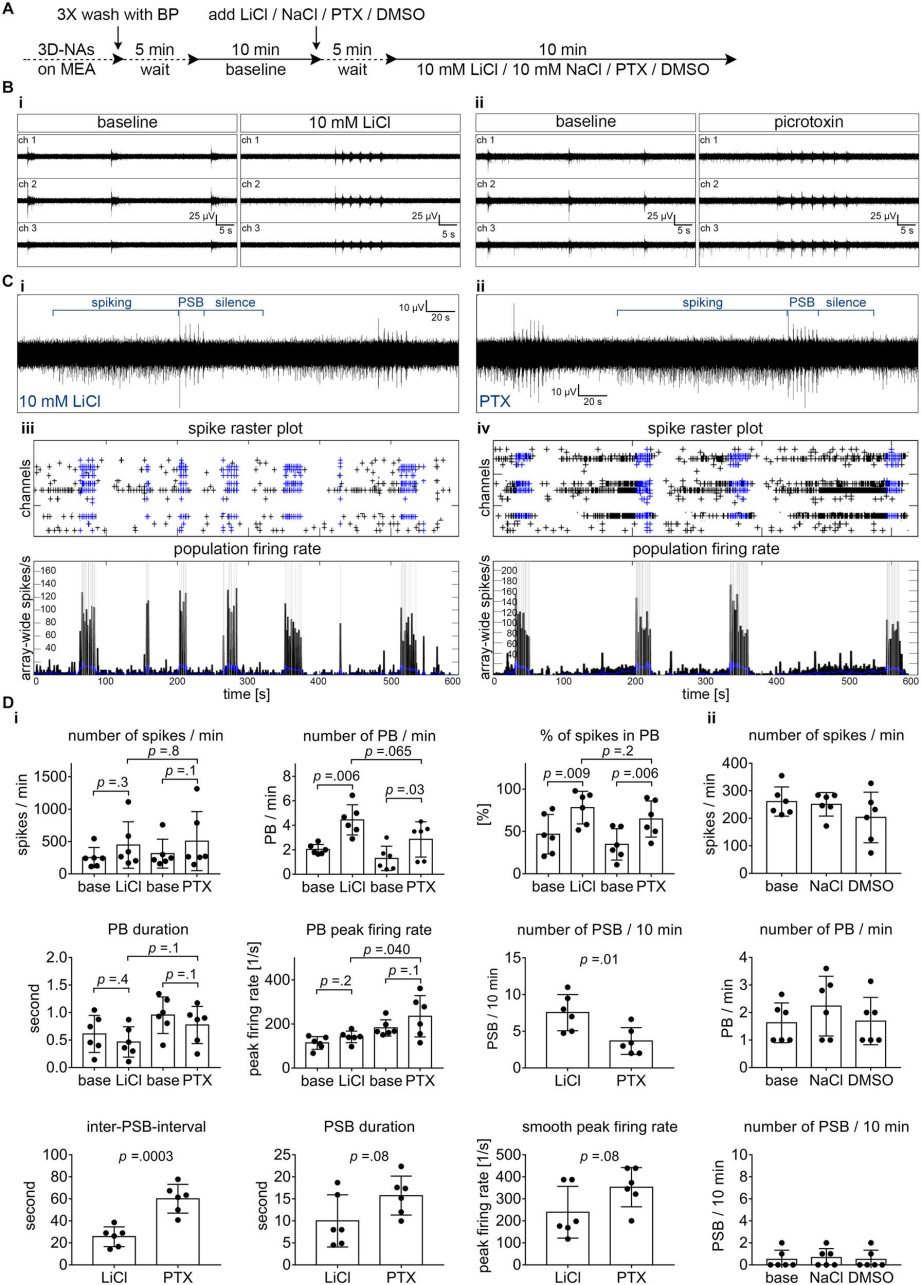
Overdose concentration of LiCl-induced epileptiform activity is comparable to GABA_A_ receptor antagonist-induced epileptiform activity in human cortical networks. **A** Schematic representation of the applied protocol to evaluate the chemically induced epileptiform activities with LiCl and picrotoxin (PTX) in the hiPSC-derived 3D-neural aggregate cultures with synchronous network activity (day 20–50 after seeding). **B** Representative MEA-recordings (three individual channels are shown) showing the properties of network activity at baseline and after the application of (**B**, **i**) 10 mM LiCl or (**B**, **ii**) 50 µM PTX. **C** Representative MEA-recordings (five-minute, one channel) showing the epileptiform activity induced by **C, i** 10 mM LiCl or **C**, **ii** 50 µM PTX. The blue lines are marking the different stages of epileptiform activities: the population super-bursts (PSB) are proceeded by prominent spiking and are followed by a period of complete inactivity or silence. **C** Spike raster plots and population firing rate diagrams showing the epileptiform events induced by **C**, **iii** LiCl and **C**, **iv** PTX. **D**, **i** Diagrams illustrating the neuronal network parameters in response to 10 mM LiCl and PTX. **D**, **ii** Diagrams showing no change in network properties in the corresponding control experiments with equimolar NaCl and DMSO. *n* = 6, *N* = 2. The data is presented as mean ± standard deviation. A two-tailed, paired or unpaired *t* test was applied to calculate the shown *p* values.

This LiCl_overd._-induced population super bursting reminded us of seizure events observed in epileptic patients^16^ and chemically induced seizures in animal models of epilepsy^17^. In order to examine more closely, if LiCl_overd_.-induced population super bursting represented seizure-like activity of human cortical neurons in vitro, we compared network firing of human cortical neurons exposed either to overdose concentrations of LiCl or to picrotoxin, a GABA_A_-receptor antagonist commonly used to induce seizure activity in in vitro^18^ and in vivo^17^ animal models (Fig. 2).

For this purpose, we exposed human neurons cultured on MEAs either to overdose concentrations of LiCl or to 50 µM GABA_A_-receptor antagonist picrotoxin. Control measurements were conducted to confirm that LiCl_overd._-induced abnormal activity is reversible (Supplementary Fig. 6A, C), and cannot be induced by equimolar sodium chloride (10 mM, NaCl) and DMSO (endc. 0.1%) (Fig. 2D, ii).

Interestingly, also picrotoxin caused a transition from synchronously active neuronal networks into neuronal networks having population super bursting (Fig. 2B, C) and partially synchronous networks turned into highly synchronous network activity (Supplementary Fig. 7A, B, Supplementary Fig. 10). In a pilot experiment, we tested pico, nano, and micromolar concentrations of picrotoxin and observed that Picrotoxin concentrations >10 µM caused consistently increased population bursting (Supplementary Fig. 10).

Comparative analysis revealed differences between LiCl_overd._-induced and picrotoxin-induced population super bursting properties recorded from initially synchronously active human neuronal networks (Fig. 2D). In contrast, the properties of population bursting in LiCl_overd._-induced and picrotoxin-induced increased-population bursting of initially partial-synchronously active neuronal networks do not show major differences (Supplementary Fig. 7C). In addition, we noticed that increased synchronous neuronal network activity partially or fully remained after washout of picrotoxin (Supplementary Fig. 6B, D).

These data demonstrate that acute application of overdose concentrations of Lithium causes abnormal neuronal network activity patterns in human iPSC neuronal circuits resembling chemically induced epileptiform-like activity.

### Lithium increases neuronal excitability

**Fig. 3:**
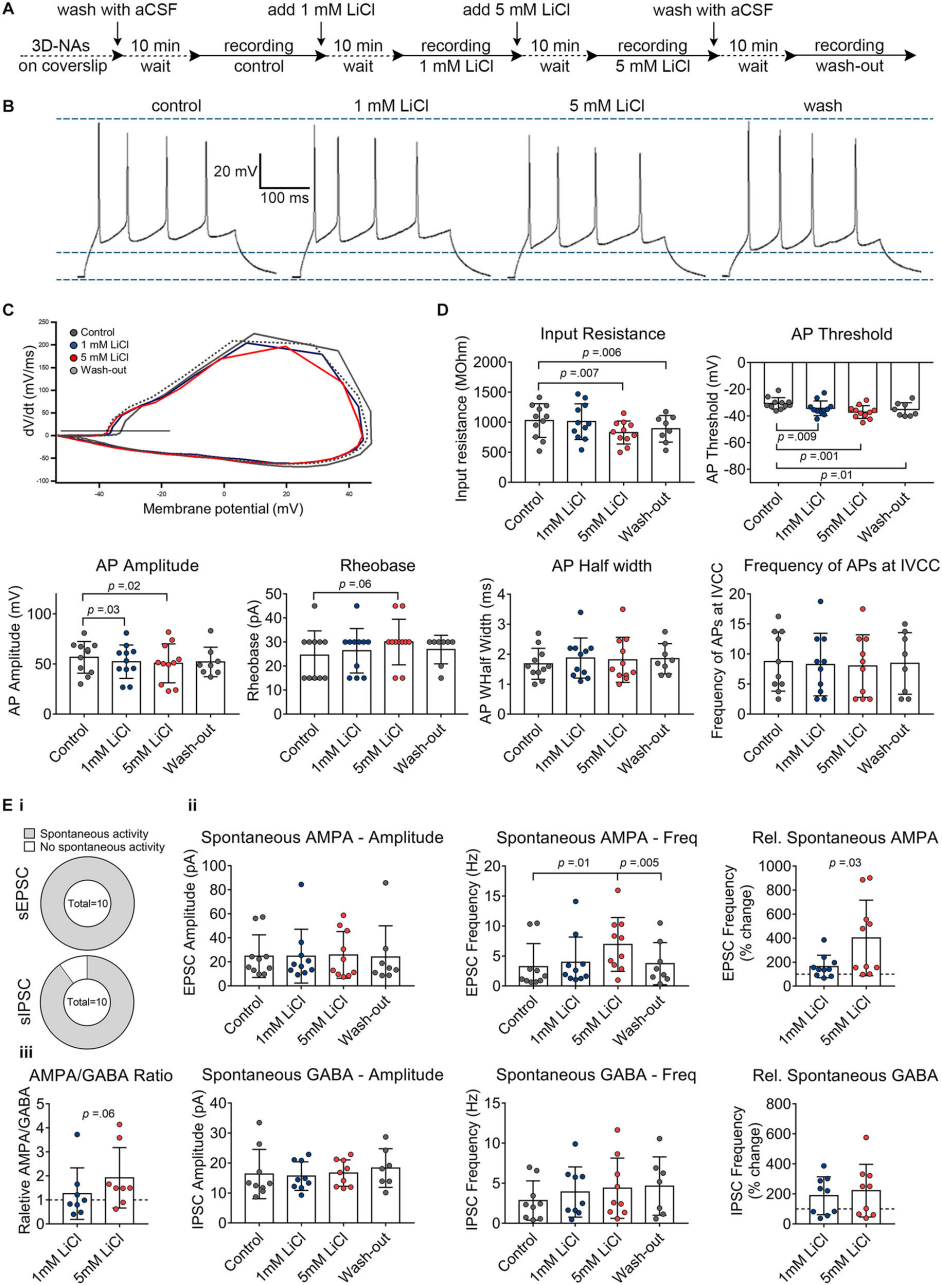
Patch–clamp recordings reveal the concentration-dependent impact of lithium chloride (LiCl) on excitability and synaptic function of human cortical neurons. **A** Schematic representation of the applied protocol to evaluate the effect of therapeutic and overdose concentrations of LiCl on the hiPSC-derived neurons. **B** Examples of evoked firing responses to a 300 ms current injection of 45 pA into a current-clamped neuron at baseline, exposed to 1 mM, 5 mM LiCl, and after wash-out. **C** Examples of phase plane plots, calculated as the first derivative of the membrane potential at the initial AP, in a train of at least two APs, against the membrane potential. The black line denotes the AP threshold (at 10 mV/ms). **D** Diagrams illustrate the membrane potential parameters in the different conditions with individual cells shown as scattered dots. *n* = 11 (*n* = 8 for wash-out), *N* = 3. **E**, **i** Pie charts show the percentage of cells with spontaneous EPSCs and spontaneous IPSCs. A total number of cells is given in the center. **E**, **ii** Average frequency and amplitude of spontaneous EPSCs and IPSCs in the different conditions with individual cells shown as scattered dots. The relative frequency of the spontaneous events compared to baseline is also shown. **E**, **iii** The ratio of the average frequency of spontaneous AMPA/GABA, *n* = 10, *N* = 3. The experiments were done in a paired setup. The data are presented as mean ± standard deviation. One-way ANOVA with Dunnett’s correction (baseline compared to the indicated group) was applied to calculate the shown *p* values. For comparing the “wash-out” group, a paired *t* test was used for *p* value calculation. In the diagrams showing relative values, the *p* values were calculated by unpaired *t* test.

To obtain insights into a prospective mode-of-action of therapeutical and overdose concentrations of lithium on human neurons, we applied whole-cell voltage- and current–clamp recordings to assess the passive membrane, excitability, and synaptic properties of human iPSC neurons acutely exposed to LiCl_thera_ and LiCl_overd_ concentrations. The electrophysiological assessment was performed in artificial cerebrospinal fluid (ACSF) without LiCl, followed by a consecutive wash in of 1 mM (LiCl_thera_) and 5 mM LiCl (LiCl_overd_), and finally wash-out of LiCl (Fig. 3A).

When injecting currents at stepwise increments with the cell in current–clamp, 11 out of 11 (control), 11 out of 11 (LiCl_thera_), and 11 out of 11 (5 mM LiCl_overd_) neurons showed evoked responses, i.e., APs (Fig. 3B). From these APs we constructed phase-plane plots (Fig. 3C) to calculate the AP threshold, AP amplitude, and AP half-width. While AP half-width and frequency of evoked APs did not show differences between control, LiCl_thera_- and LiCl_overd_-treated groups, a lithium concentration-dependent decrease of the AP amplitude was observed (Fig. 3C). Moreover, LiCl_thera_ and LiCl_overd_ enhanced neuronal excitability of neurons indicated by a reduction (i.e., more hyperpolarized) of the AP threshold from 30.6 ± 1.3 mV (control) to 34.2 ± 1.7 mV (LiCl_thera_) and further to 37.1 ± 1.5 mV (LiCl_overd_). LiCl_overd_-treated neurons showed a significant reduction of the input resistance (1029 ± 84.7 MOhm (control) vs. 828.4 ± 57.8 MOhm (LiCl_overd_), possibly explaining the increased Rheobase (24.6 ± 3.0 pA (control) vs. 30.0 ± 2.9 pA (LiCl_overd_)) despite the reduction in AP threshold (Fig. 3D).

Whole-cell voltage-recordings revealed that neither LiCl_thera_, nor LiCl_overd,_ change the amplitude or frequency of GABA currents (Fig. 3E, iii), indicating that LiCl_overd_-induced epileptiform activity pattern observed by MEA recordings is not mediated by LiCl_overd_-induced modulation of GABA_A_-receptor activity. Interestingly, only LiCl_overd_ caused a significant and remarkable increase in spontaneous AMPA-receptor current frequency, without influencing the amplitude of AMPA-currents (Fig. 3E, ii).

After wash-out of LiCl, input resistance and AP threshold remained slightly decreased (Fig. 3D, comparison control and wash-out). However, MEA recordings revealed that after wash-out no alterations before and after Lithium-treatment are detectable at neuronal network level (Supplementary Figs. 4 and 6).

By combining the whole-cell voltage– and current–clamp recording data, we conclude that LiCl_thera_ and LiCl_overd_ enhance neuronal excitability. However, the increased neuronal excitability seems specific for glutamatergic neurons since LiCl_overd_ increased spontaneous AMPA-receptor mediated transmission, but did not change the spontaneous GABA-receptor mediated transmission.

### Perampanel suppresses lithium overdose-induced epileptiform-like activity in human iPSC-cortical circuits

**Fig. 4:**
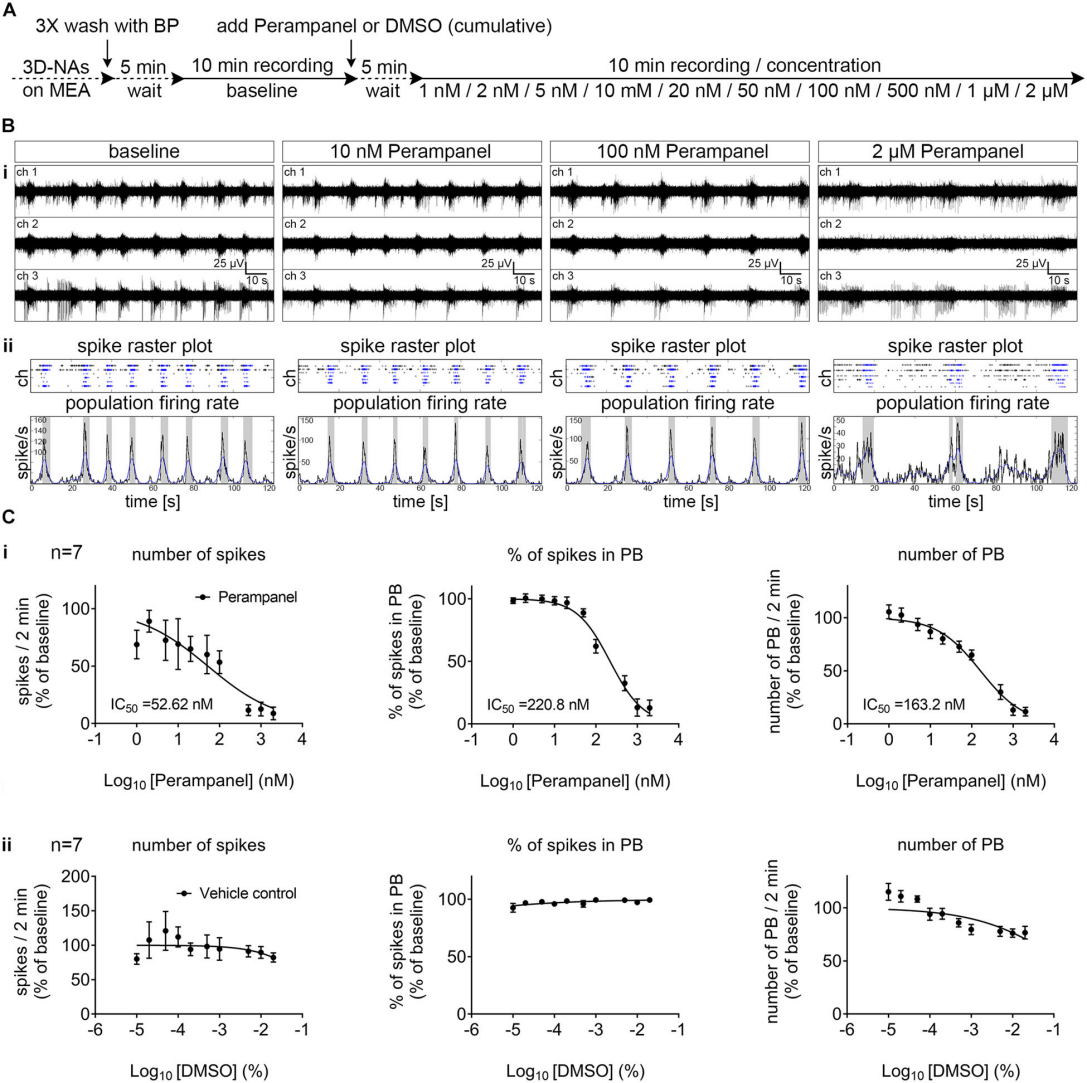
Perampanel suppresses the synchronous neuronal activity in concentration-dependent manner. **A** Schematic representation of the applied protocol to evaluate the cumulative dose–response of hiPSC-derived highly synchronous neuronal networks (day 30 after seeding) to the AMPA-receptor inhibitor Perampanel. **B**, **i** Representative examples of MEA-recordings (three individual channels are shown) with their corresponding. **B**, **ii** Spike raster plot and population firing rate diagrams showing the suppression of synchronous population bursting by increasing concentrations of Perampanel. **C**, **i** Dose–response curves showing the suppression of network parameters (number of spikes, % of spikes organized in population bursts-PB and number of PB) with increasing concentrations of Perampanel. The data is shown as % of change compared to baseline and represents the mean ± standard error of the mean values. The IC_50_ values are given, *n* = 7, *N* = 2. **C**, **ii** Dose–response curves showing the stable network activity parameters with the application of DMSO vehicle control, *n* = 7, *N* = 2.

Since seizures represent a severe side-effect observed in lithium-treated patients^3,19,20^, we aimed to evaluate a pharmacological intervention approach that may suppress pathological LiCl_overd_-induced human neuronal network function. We examined if the AMPA-receptor antagonist Perampanel could suppress LiCl_overd_-induced seizure-like activity in hiPSC-cortical neurons. In contrast to another AMPA-receptor antagonist, e.g., CNQX or NBQX, Perampanel is the only AMPA-receptor antagonist that crosses the blood–brain barrier and is the first-in-class medication to treat partial seizures and generalized tonic-clonic seizures in patients older than 12 years^21^. Since the potency assessment of Perampanel, i.e., dose–response curves of Perampanel, has only been analyzed in animal-based model systems^22,23^ or frog oocytes microinjected with human brain membrane preparations^24^, we first evaluated the effective concentration range of Perampanel alone on human neuronal function and performed MEA-recordings in synchronously active human iPSC-cortical neuronal cultures (30 DIV, 80 DIV) consecutively exposed to increased Perampanel concentrations (Fig. 4A, Supplementary Fig. 8A). We revealed that Perampanel causes a dose-dependent reduction in the number of spikes, number of PB, and percentage of spikes organized in PB. Depending on the used neuronal network parameters to calculate the IC_50_-value, the IC_50_-value of Perampanel in human in vitro neurons were between 55 and 220 nM (Fig. 4C, Supplementary Fig. 8).

**Fig. 5:**
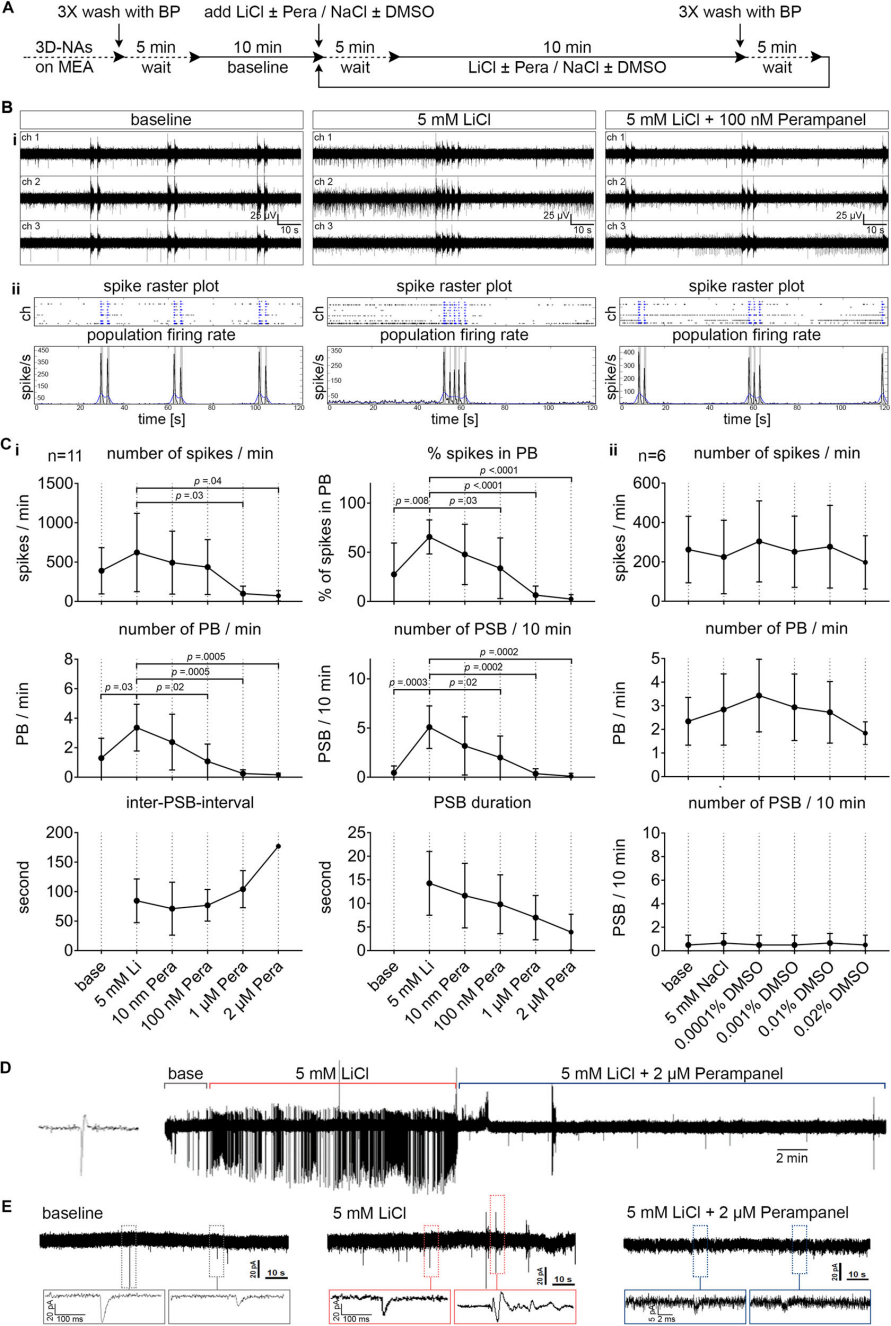
Perampanel counteracts LiCl-induced epileptiform activity in human cortical networks. **A** Schematic representation of the applied protocol to evaluate the applicability of Perampanel to reverse LiCl-induced epileptiform activity in human iPSC-derived synchronously active cortical networks (day 20–50 after seeding). **B**, **i** Representative examples of MEA-recordings (three individual channels are shown) with their corresponding. **B**, **ii** Spike raster plot and population firing rate diagrams showing the suppression of LiCl-induced epileptiform population super-bursting by Perampanel. **C**, **i** The suppression of network parameters with increasing concentrations of Perampanel. The data are presented as mean ± standard deviation, *n* = 11, *N* = 2. One-way ANOVA with Tukey’s correction (comparison between the indicated groups) was applied to calculate the shown *p* values. **C**, **ii** Diagrams showing no change in network properties in the corresponding control experiments with equimolar NaCl and DMSO, *n* = 6, *N* = 2. **D** Cell-attached patch–clamp recording of human iPSC-derived cortical neuron, showing the increased spontaneous activity after the application of overdose concentration of LiCl (5 mM) and its suppression by Perampanel (2 µM). **E** Whole-cell patch–clamp traces showing the increased frequency of spontaneous EPSCs after the application of overdose concentration of LiCl (5 mM) and its suppression after the application of Perampanel (2 µM). The boxes mark regions of interest showed at higher magnification below. PB population burst, PSB population super burst.

Next, we evaluated if inhibiting AMPA-receptor activity by Perampanel was able to suppress 5 mM LiCl_overd_-induced epileptiform neuronal network activity (Fig. 5). Based on the pre-evaluated IC_50_ values, we selected a concentration range for Perampanel (10 nM, 100 nM, 1 µM, 2 µM). Perampanel application led to a dose-dependent reduction in LiCl_overd_-induced epileptiform neuronal network activity (Fig. 5B, C). We observed that 100 nM Perampanel application resulted in a neuronal population activity that had comparable properties as measured before LiCl_overd_ application. In detail, the application of 100 nM Perampanel was sufficient to counteract the LiCl_overd_-mediated increase in a number of spikes, number of PB, percentage of spikes organized in PB, and reduction of PSB (Fig. 5C, i, compare values for the base with values after 100 nM Perampanel treatment). However, the most effective reduction in the number of population super bursting was achieved by Perampanel concentrations >1 µM (Fig. 5C, i). In additional experiments using 10 mM LiCl, we observed a comparable reduction of LiCl_overd_-epileptiform-like activity by Perampanel (Supplementary Fig. 9). Cell-attached and whole-cell recordings confirm that Perampanel prevented LiCl_overd_-induced hyper spiking activity (Fig. 5D) and reduced spontaneous AMPA-receptor mediated transmission (Fig. 5E).

These data demonstrate that a clinically applicable AMPA-receptor antagonist reduces epileptiform-like activity in human iPSC neurons exposed to overdose concentrations of lithium chloride.

## Discussion

By applying MEA and patch–clamp technology on a human iPSC-derived cortical in vitro model, we provide insights into the immediate influence of therapeutically effective and overdose concentrations of lithium on a human synaptic, single neuron, and neuronal network function. We demonstrate that therapeutically effective concentrations of lithium increase neuronal excitability of human neurons and lead to increased network activity. However, overdose concentrations of lithium (>1 mM), known to cause severe side-effects in human, e.g., seizures, change the neuronal activity of in vitro human cortical neurons into a seizure-like activity pattern, that is similar to neuronal network activity pattern elicited by commonly seizure-causing compounds, e.g., gabazine and picrotoxin.

The mechanisms behind the net increased network excitability by lithium are likely complex. In this study, we provide new evidence that lithium reduces the AP threshold. This effect is interesting and warrants further mechanistic studies of isolated sodium and potassium currents. We also show that overdose concentrations of lithium reduce the input resistance, an effect that is expected to counteract the increased excitability caused by the reduction in the AP threshold. Part of the decreased input resistance could be explained by an increased GIRK basal current^25^. This study on rodent hippocampal neurons^25^ also showed that lithium attenuated neurotransmitter-evoked GIRK currents, which, on the other hand, would be expected to increase network excitability. Consistent with our findings of increased PB by lithium, Butler–Munro et al.^26^ have shown in rodent olfactory neurons that lithium decreases the AP afterhyperpolarization. We show that 5 mM lithium substantially increased the frequency of spontaneous AMPA receptor-mediated EPSCs, while not significantly increasing the frequency of spontaneous GABA_A_-mediated IPSCs. Spontaneous synaptic currents consist of a mixture of AP-dependent release and AP independent release of transmitter. Since we found a net increase in excitability it is likely that the increased frequency of AMPA receptor-mediated EPSCs is mostly explained by an increased frequency of spontaneous APs in glutamatergic neurons. We can, however, not exclude that lithium also increases the release probability at glutamatergic synapses. Importantly, however, our result shows that overdose concentrations of lithium increase the excitatory to the inhibitory ratio in the human cortical cultures, which should profoundly contribute to the increased network excitability. This result also suggests that the net excitability is increased in glutamatergic neurons, whereas it is not changed in GABAergic neurons. We did not detect any change in the amplitude of the spontaneous AMPA receptor-mediated EPSCs by our overdose concentration of lithium (5 mM), indicating no direct effect on AMPA receptor function. Higher concentrations of lithium have, however, been shown to directly affect AMPA receptor function. For instance, Gebhardt and Cull-Candy^27^ used 10 mM LiCl and showed increased AMPA receptor function in hippocampal neurons, likely because of decreased desensitization.

We show that the epileptiform-like activity induced by overdose concentration of lithium in the human cortical culture was counteracted by the antiepileptic drug perampanel (Fycompa^™^). Perampanel is an anti-epileptic drug that is used to treat partial seizures and generalized tonic-clonic seizures^28^ and is the only AMPA receptor antagonist accepted for clinical use. Since animal-based in vitro and in vivo models are considered of limited translational medical value, our human iPSC-derived neuronal in vitro model represents a valuable alternative to study further the functional impact of neuropsychological and anti-epileptic drugs.

In a review about brain oscillations in lithium-induced changes of brain function, the author speculated “that lithium might enhance cortical excitability acutely”^29^, which has not yet been analyzed on human neurons. It was thus unknown, how lithium directly affects human neuronal function at the synaptic, individual neuron, and network level. Previous works on the effect of lithium on the electrophysiological function of human iPSC neurons have focused on the chronical effects. For instance, the human iPSC-neuronal electrophysiological function was assessed after 3 days^30^, 1 week^9^, or 2 weeks^8^ exposure to lithium. Mertens et al.^9^ showed that chronical lithium application (1 week) decreases spontaneous AP frequency in human iPSC-neurons. Darville et al.^30^ applied calcium imaging on Fluo-4 loaded human iPSC-derived cortical and showed that chronical lithium application (3 days, 0.5 mM lithium) causes an increase of calcium events. In our study, we focused on the acute (within minutes) effects of lithium on human cortical networks, which has implications for the acute overdose effects of lithium in patients.

Mimicking the acute concentration-dependent bi-modulatory impact of lithium chloride on the human neuronal network function in vitro, required a highly functional neuronal network comprising functional interconnected inhibitory and excitatory neurons. We demonstrate that our human iPSC-derived cortical in vitro model is suitable to mimic drug-induced epileptiform-like activity in human neurons. In contrast, previous works failed to induce synchronous bursting in human iPSC-derived neurons by using GABA-A receptor antagonists^31,32^. In addition, analyzing the number of all recorded spikes detected by all electrodes is a common approach in the neuro-human iPSC-research field^33–35^. However, here we show that this neuronal parameter is insufficient to reveal the impact of neuropharmacological compounds on human neuronal network activity.

### Implications for future studies and discussion about the prospective clinical relevance

Following the good results of Perampanel in the treatment of patients with refractory partial-onset seizures and generalized tonic-clonic seizures, further indications for other types of epilepsy could be examined. An extension of use to other forms of epilepsies might be very interesting, however, this requires proof of concept studies demonstrating efficacy in humans. As a first step, drug testing in appropriate preclinical model systems is essential. Here, we showed that Perampanel suppresses epileptiform-like activity in human neuronal circuits exposed to overdose concentrations of lithium. Moreover, we showed that human neuronal circuits exposed to Perampanel did not become totally inactive, but show a concentration-dependent reduction in the number and power of synchronous neuronal discharges.

Potency assessment of Perampanel in our human iPSC-neuronal in vitro model revealed an IC_50_ between 55 and 220 nM, which is comparable with previous in vitro data obtained from cultured rat hippocampal neurons (IC_50_: 300 nM where 10 µM AMPA were used^22^), cultured rat cortical neurons (IC_50_: 53–90 nM^36^) and rat hippocampal slices (IC_50_: 230 nM^37^). It is worth mentioning that measurements of AMPA-receptor currents by whole-cell patch recordings applied on frog oocytes where membrane preparations obtained from human brain samples were microinjected showed an IC_50_ for Perampanel in the range of 4.3–7.2 µM^24^. However, clinical trials and recent patient data indicate that the effective Perampanel concentration is in the nanomolar range. In detail, data obtained from the clinical trial phase 3 showed that patients treated efficiently with Perampanel show a blood concentration in the range from 212 to 358 ng/mL (0.61–1.01 µM, with a 4 mg daily dose) and from 275 to 456 ng/mL (0.79–1.31 µM, with a 6 mg daily dose)^38,39^. Recent clinical data showed an even broader range of required blood levels of Perampanel to achieve effectiveness in patients^40,41^. Since more than 90% of Perampanel is bound by proteins^38,39^, the free Perampanel blood concentration is in the nanomolar range indicating that human brain AMPA receptor activity must be effectively modulated by Perampanel concentration in the nanomolar range. Thus, potency assessment of Perampanel in our human iPSC neuronal model is in line with data obtained from in vitro animal models and clinical efficacy data.

The present data clearly is in support of further clinical development of Perampanel (Fycompa^™^) into other forms of epilepsy. The here presented proof-of-concept enables the identification of other anti-epileptic drugs (e.g., valproate) and their potency in suppressing lithium-overdose-induced epileptiform-like activity in a human functional neuronal in vitro model.

## Supplementary information

Suppl. Table 1: Antibody list

Suppl.Figure 1 | Generation and properties of human-specific highly functional cortical circuits from human iPSC (human neuronal sensor chips).

Suppl. Figure 2 | Cortical layer-specific and parvalbumin-neurons in human 3DNA cultures

Suppl. Figure 3 | Acute application of therapeutic and overdose concentrations of LiCl on human iPSC-derived neuronal networks

Suppl. Figure 4 | Quantitative assessment of acutely applied therapeutic and overdose concentrations of LiCl on human iPSC-derived neuronal networks

Suppl. Figure 5 | Impact of lithium chloride (LiCl) on partial synchronous human cortical networks recorded by MEAs.

Suppl. Figure 6 | The epileptiform activity induced by overdose concentration of lithium chloride (LiCl) is reversible in synchronous and partial synchronous human cortical networks.

Suppl. Figure 7 | Chemically induced epileptiform activity in partial synchronous human cortical networks.

Suppl. Figure 8 | Perampanel suppresses the synchronous neuronal activity in a concentration-dependent manner (additional experiments).

Suppl. Figure 9 | Perampanel counteracts 10 mM LiCl-induced epileptiform activity in human cortical networks (additional experiments).

Suppl. Figure 10 | Picrotoxin dose-response assessment in human iPSC-cortical neuronal networks.

## Data Availability

The data that support the findings of this study are available from the corresponding author, upon reasonable request.
