# Supplementary material for: Differential acute impact of therapeutically effective and overdose concentrations of lithium on human neuronal single cell and network function
Source: Transl Psychiatry. 2021 May 12;11:281. doi: 10.1038/s41398-021-01399-3 (PMC8115174; doi:10.1038/s41398-021-01399-3)
Supplement: Supplementary file 1 — Suppl. Table 1: Antibody list [file 41398_2021_1399_MOESM1_ESM.pdf]

Antibody	Supplier (cat. No.)	Host	Dilution
bIII-Tubulin	R&D (MAB1195)	mouse	1:2000
CTIP2	Abcam (ab18465)	rat	1:300
MAP2AB	Abcam (ab11267)	mouse	1:1000
MAP2AB	Abcam (ab5392)	chicken	1:2000
PSD95	Abcam (ab18258)	rabbit	1:1000
PV	Sigma-Aldrich (P3088)	mouse	1:500
SATB2	Abcam (ab51502)	mouse	1:300
S100β	Dako Agilent (Z0311)	rabbit	1:500
TBR1	Abcam (ab31940)	rabbit	1:300
VGlut1	Synaptic systems (135303)	rabbit	1:1000
Alexa Fluor 488	Thermo Fisher (A11029)	goat anti-mouse	1:500
Alexa Fluor 488	Thermo Fisher (A21208)	donkey anti-rat	1:500
Alexa Fluor 555	Thermo Fisher (A32932)	goat anti-chicken	1:500
Alexa Fluor 555	Thermo Fisher (A31572)	donkey anti-rabbit	1:500
Alexa Fluor 633	Thermo Fisher (A21071)	goat anti-rabbit	1:500
