# Supplementary figures and images for: Differential acute impact of therapeutically effective and overdose concentrations of lithium on human neuronal single cell and network function
Source: Transl Psychiatry. 2021 May 12;11:281. doi: 10.1038/s41398-021-01399-3 (PMC8115174; doi:10.1038/s41398-021-01399-3)

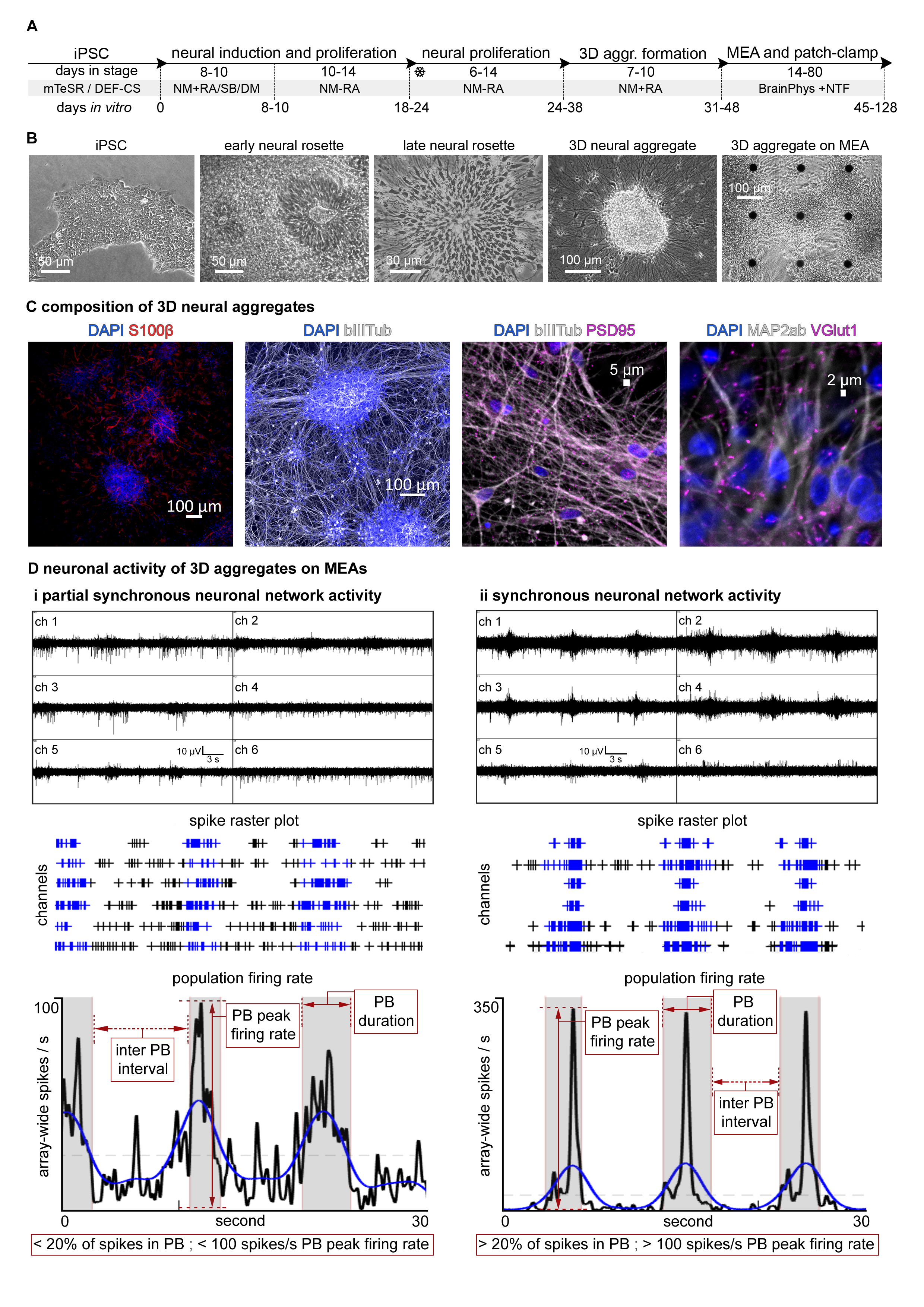

Supplement: Supplementary file 2 — Suppl.Figure 1 | Generation and properties of human-specific highly functional cortical circuits from human iPSC (human neuronal sensor chips). [file 41398_2021_1399_MOESM2_ESM.tif]

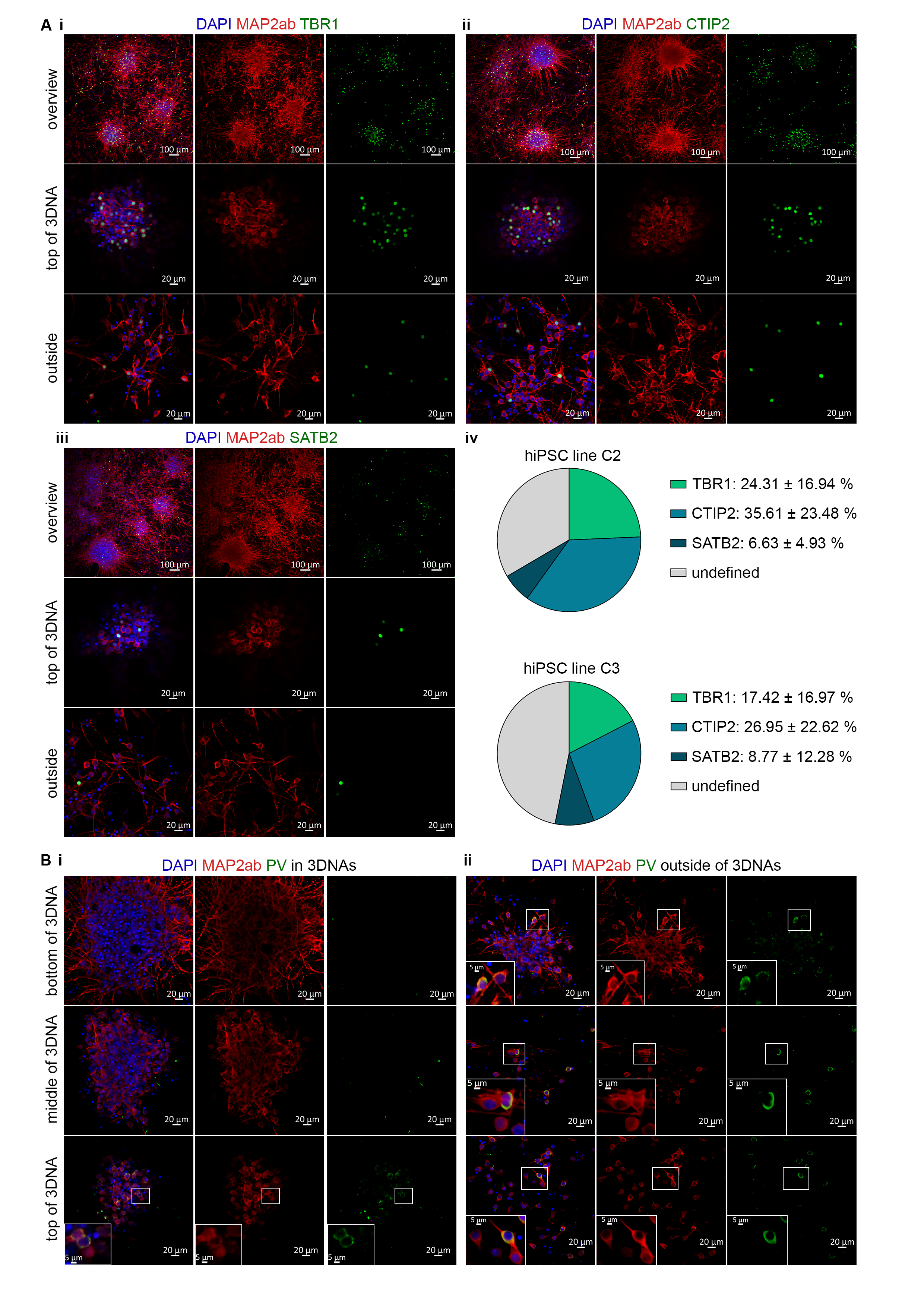

Supplement: Supplementary file 3 — Suppl. Figure 2 | Cortical layer-specific and parvalbumin-neurons in human 3DNA cultures [file 41398_2021_1399_MOESM3_ESM.tif]

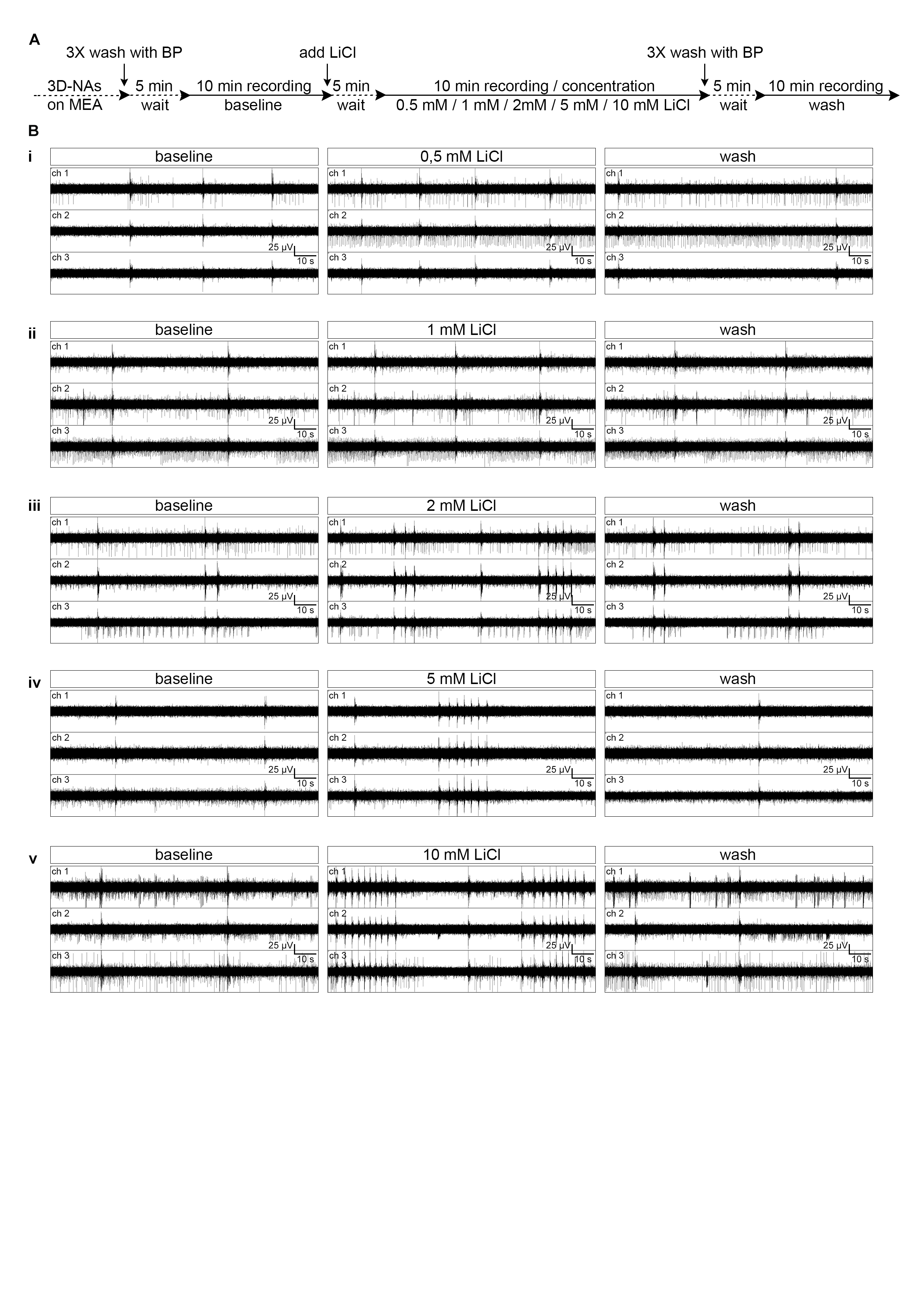

Supplement: Supplementary file 4 — Suppl. Figure 3 | Acute application of therapeutic and overdose concentrations of LiCl on human iPSC-derived neuronal networks [file 41398_2021_1399_MOESM4_ESM.tif]

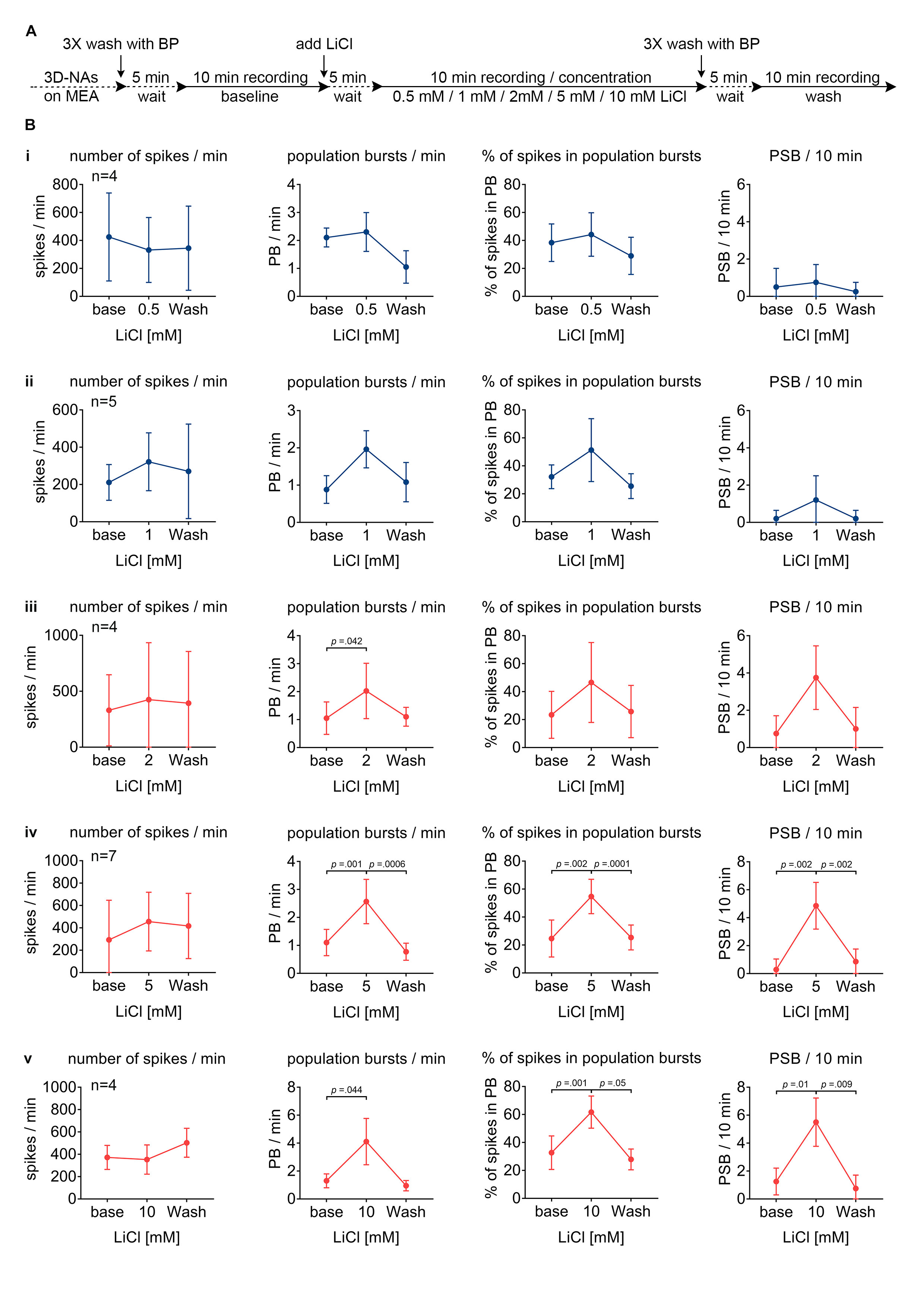

Supplement: Supplementary file 5 — Suppl. Figure 4 | Quantitative assessment of acutely applied therapeutic and overdose concentrations of LiCl on human iPSC-derived neuronal networks [file 41398_2021_1399_MOESM5_ESM.tif]

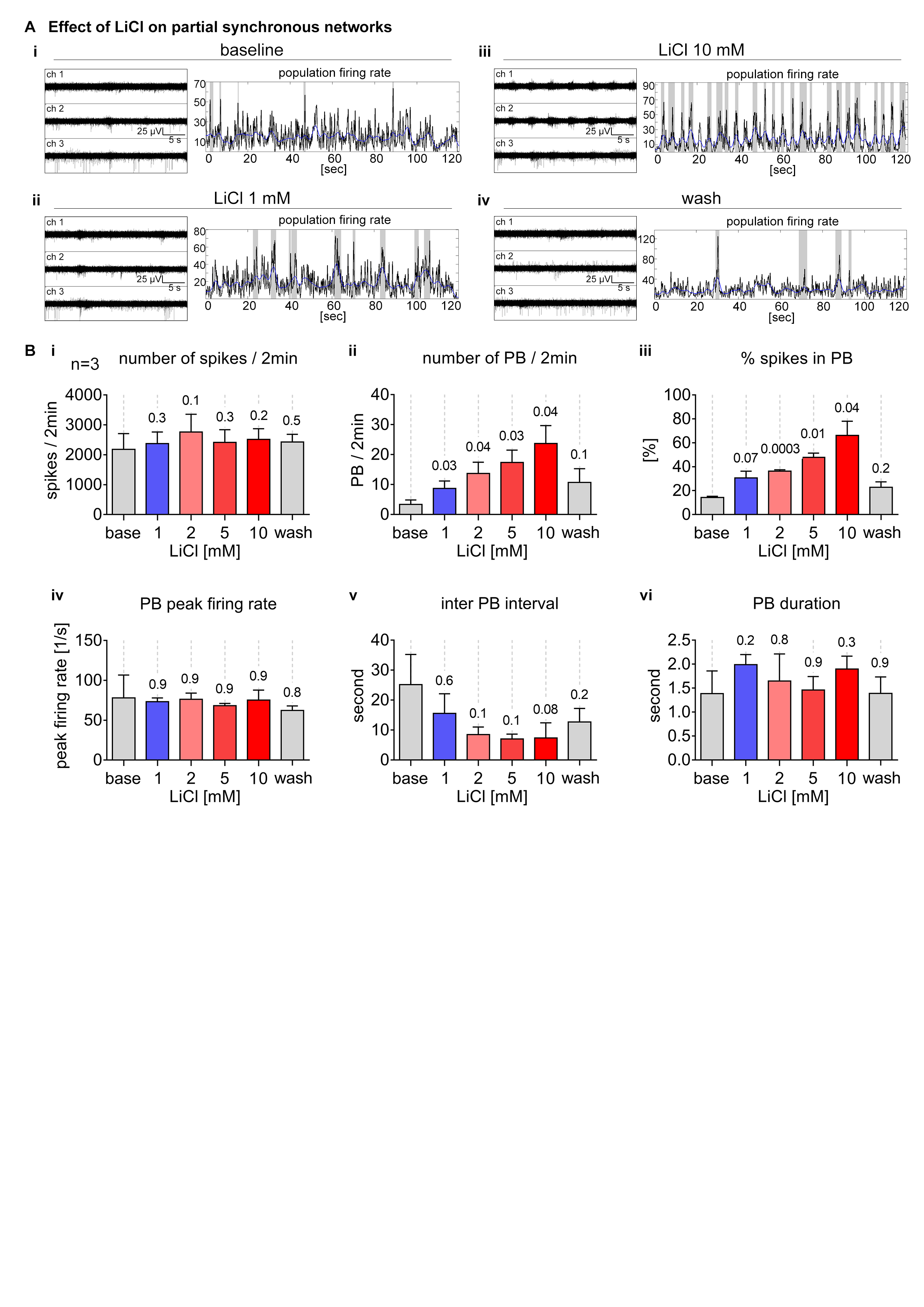

Supplement: Supplementary file 6 — Suppl. Figure 5 | Impact of lithium chloride (LiCl) on partial synchronous human cortical networks recorded by MEAs. [file 41398_2021_1399_MOESM6_ESM.tif]

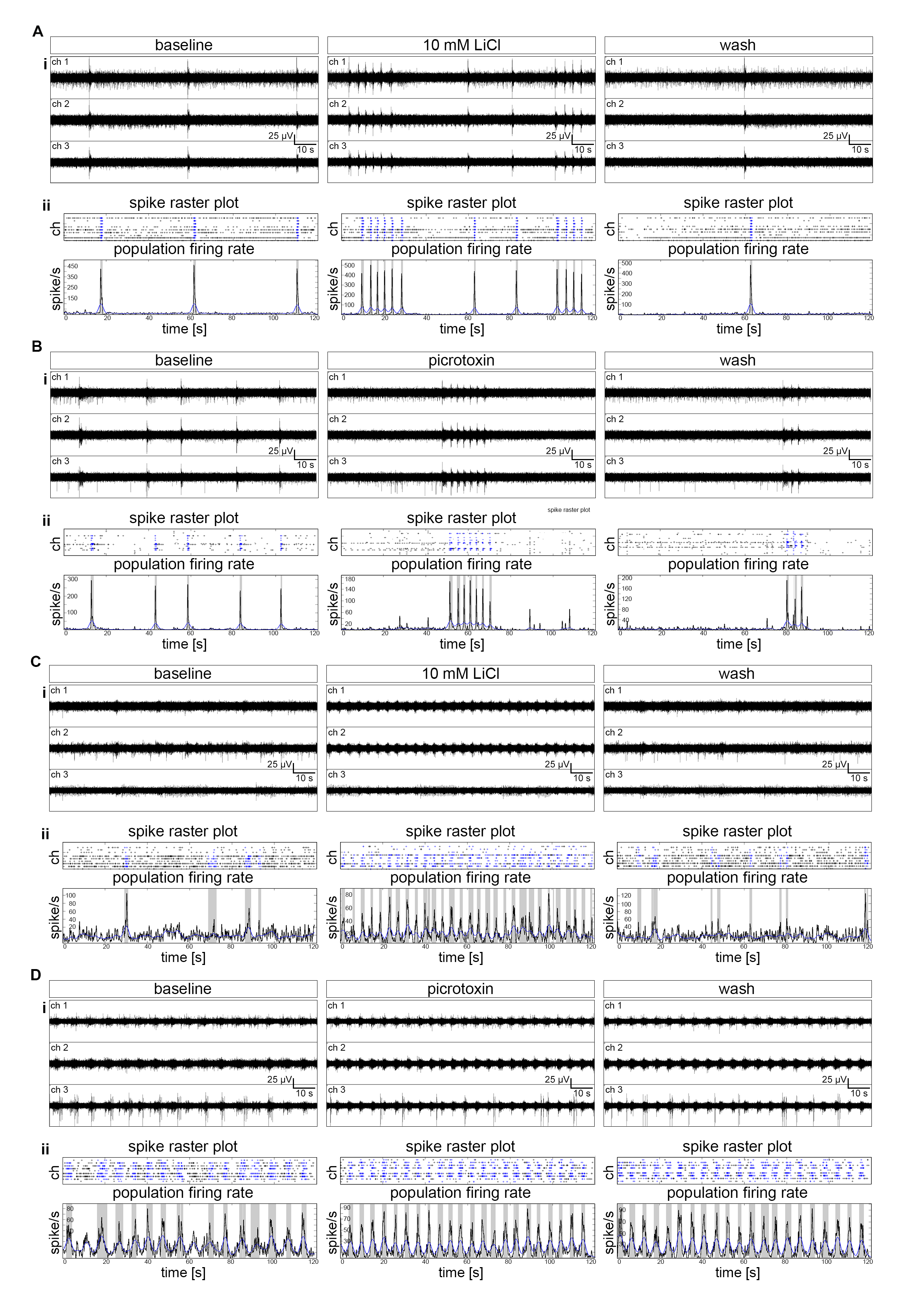

Supplement: Supplementary file 7 — Suppl. Figure 6 | The epileptiform activity induced by overdose concentration of lithium chloride (LiCl) is reversible in synchronous and partial synchronous human cortical networks. [file 41398_2021_1399_MOESM7_ESM.tif]

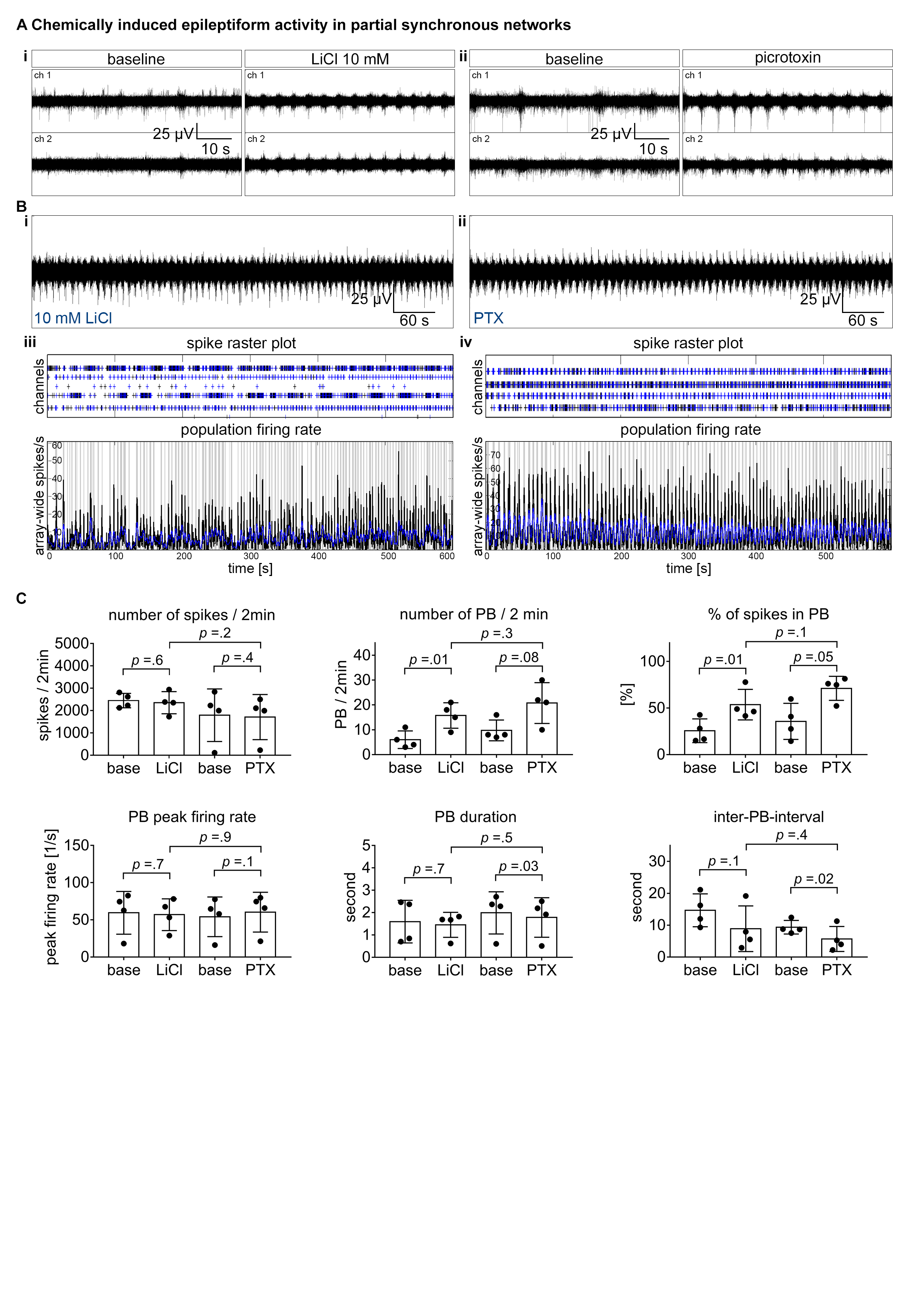

Supplement: Supplementary file 8 — Suppl. Figure 7 | Chemically induced epileptiform activity in partial synchronous human cortical networks. [file 41398_2021_1399_MOESM8_ESM.tif]

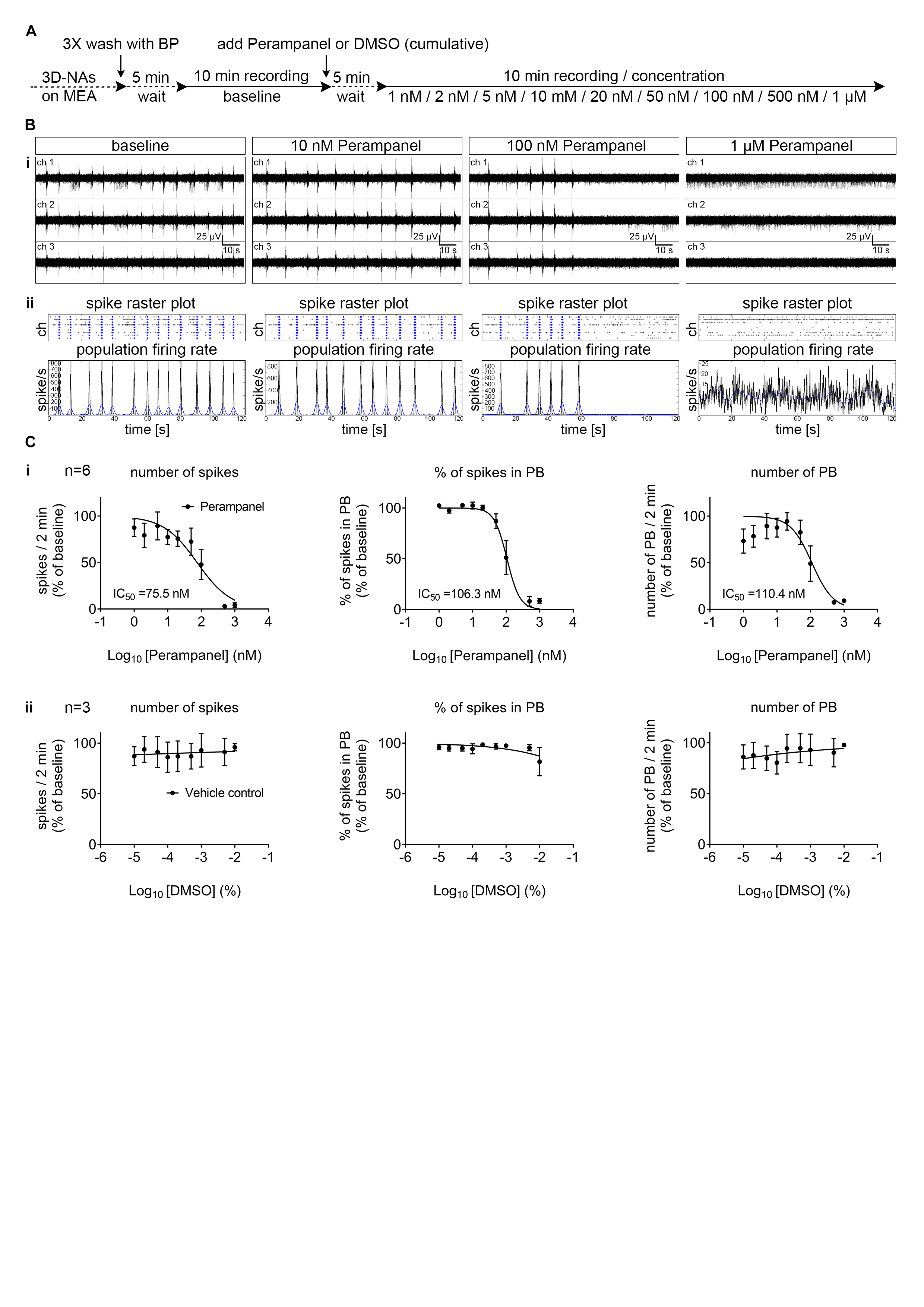

Supplement: Supplementary file 9 — Suppl. Figure 8 | Perampanel suppresses the synchronous neuronal activity in a concentration-dependent manner (additional experiments). [file 41398_2021_1399_MOESM9_ESM.tif]

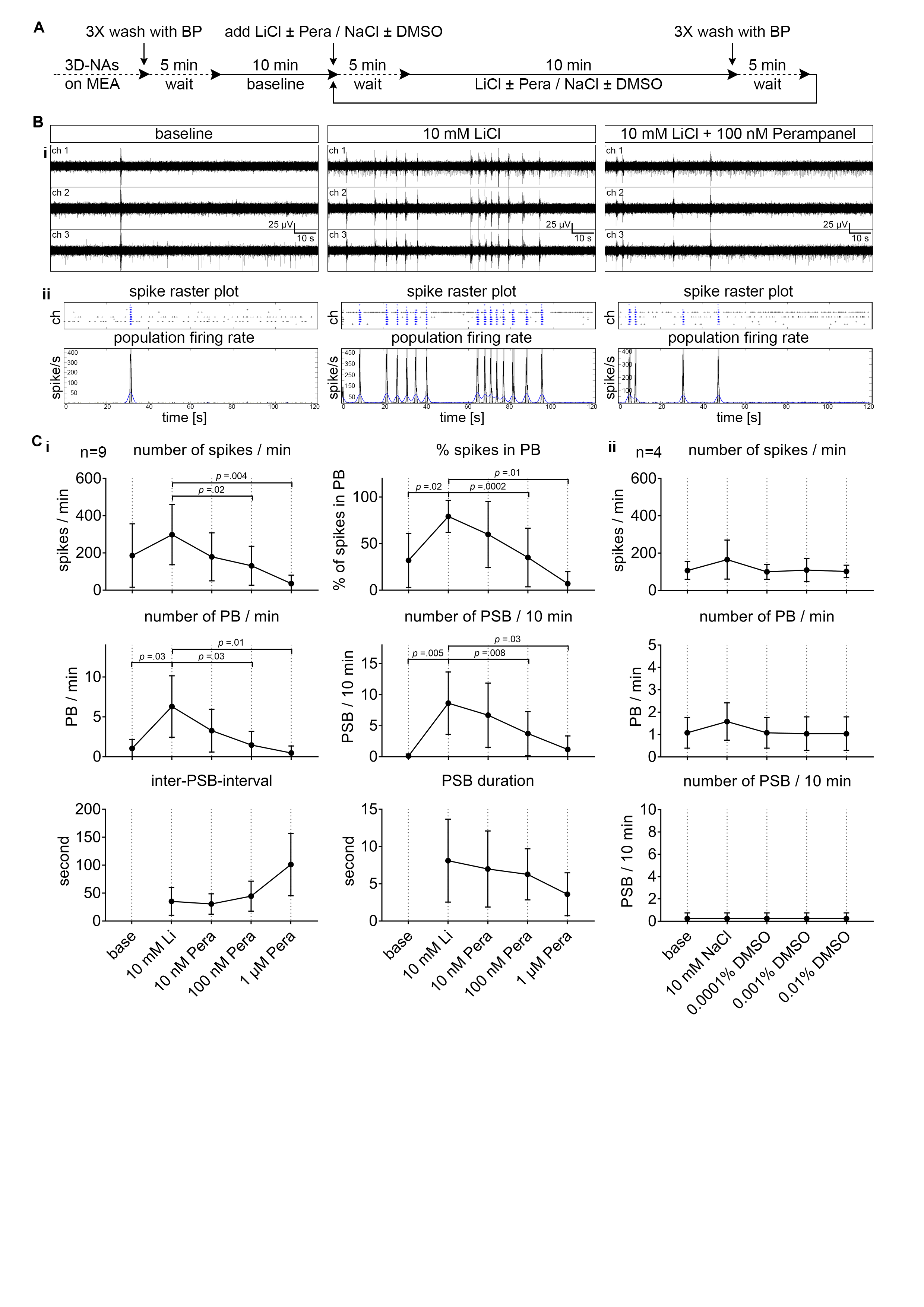

Supplement: Supplementary file 10 — Suppl. Figure 9 | Perampanel counteracts 10 mM LiCl-induced epileptiform activity in human cortical networks (additional experiments). [file 41398_2021_1399_MOESM10_ESM.tif]

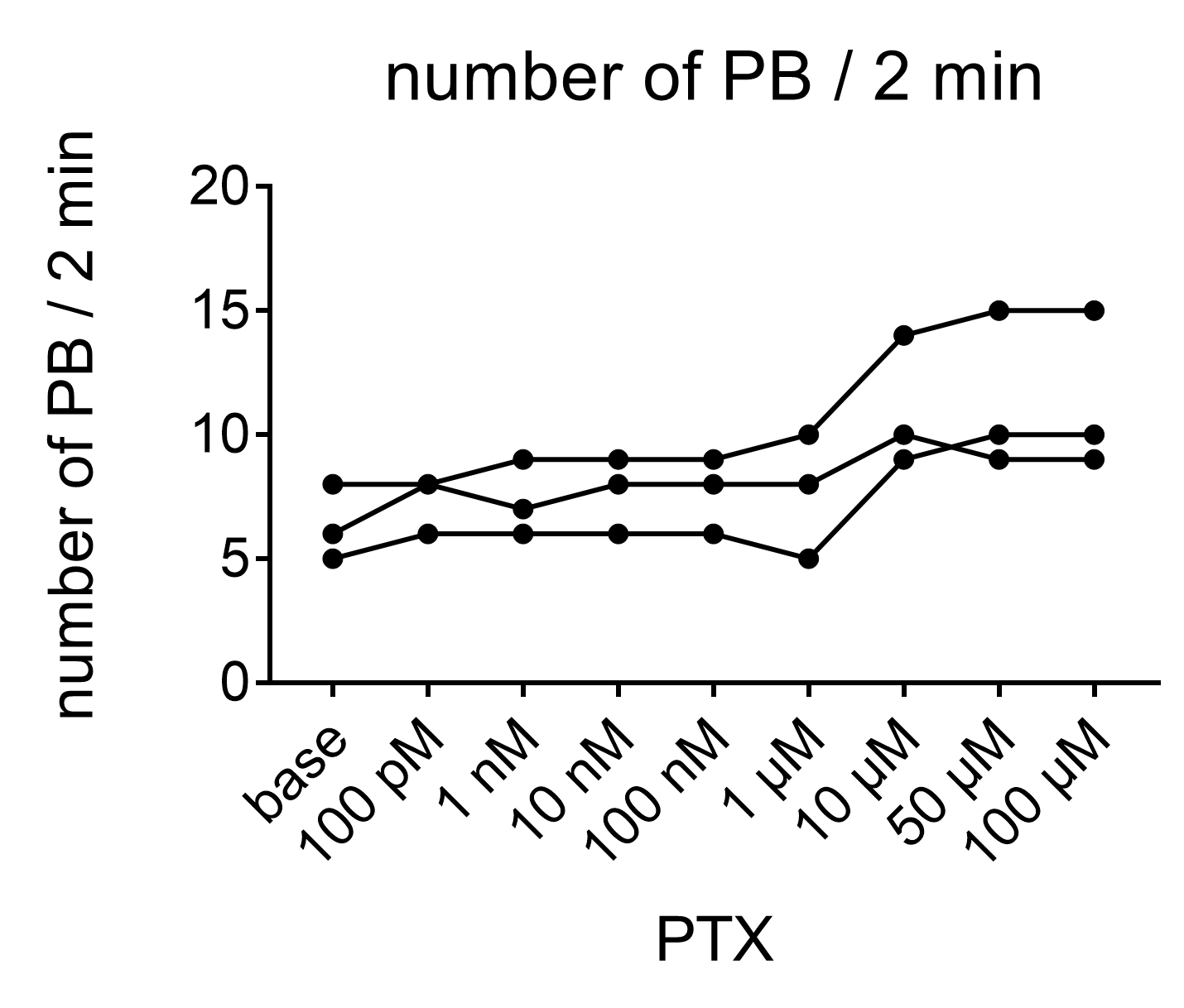

Supplement: Supplementary file 11 — Suppl. Figure 10 | Picrotoxin dose-response assessment in human iPSC-cortical neuronal networks. [file 41398_2021_1399_MOESM11_ESM.tif]
